# Relapse Prevention: An Overview of Marlatt’s Cognitive-Behavioral Model

**Published:** 1999

**Authors:** Mary E. Larimer, Rebekka S. Palmer, G. Alan Marlatt

**Affiliations:** Mary E. Larimer, Ph.D., is a research assistant professor of psychology, Rebekka S. Palmer is a graduate student in clinical psychology, and G. Alan Marlatt, Ph.D., is a professor of psychology at the Addictive Behaviors Research Center, Department of Psychology, University of Washington, Seattle, Washington

**Keywords:** AODD (alcohol and other drug dependence) relapse, relapse prevention, treatment model, cognitive therapy, behavior therapy, risk factors, coping skills, self efficacy, expectancy, AOD (alcohol and other drug) abstinence, lifestyle, AOD craving, intervention, alcohol cue, reliability (research methods), validity (research methods), literature review

## Abstract

Relapse prevention (RP) is an important component of alcoholism treatment. The RP model proposed by Marlatt and Gordon suggests that both immediate determinants (e.g., high-risk situations, coping skills, outcome expectancies, and the abstinence violation effect) and covert antecedents (e.g., lifestyle factors and urges and cravings) can contribute to relapse. The RP model also incorporates numerous specific and global intervention strategies that allow therapist and client to address each step of the relapse process. Specific interventions include identifying specific high-risk situations for each client and enhancing the client’s skills for coping with those situations, increasing the client’s self-efficacy, eliminating myths regarding alcohol’s effects, managing lapses, and restructuring the client’s perceptions of the relapse process. Global strategies comprise balancing the client’s lifestyle and helping him or her develop positive addictions, employing stimulus control techniques and urge-management techniques, and developing relapse road maps. Several studies have provided theoretical and practical support for the RP model.

Relapse, or the return to heavy alcohol use following a period of abstinence or moderate use, occurs in many drinkers who have undergone alcoholism treatment. Traditional alcoholism treatment approaches often conceptualize relapse as an end-state, a negative outcome equivalent to treatment failure. Thus, this perspective considers only a dichotomous treatment outcome—that is, a person is either abstinent or relapsed. In contrast, several models of relapse that are based on social-cognitive or behavioral theories emphasize relapse as a transitional process, a series of events that unfold over time (Annis 1986; Litman et al. 1979; Marlatt and Gordon 1985). According to these models, the relapse process begins prior to the first posttreatment alcohol use and continues after the initial use. This conceptualization provides a broader conceptual framework for intervening in the relapse process to prevent or reduce relapse episodes and thereby improve treatment outcome.

This article presents one influential model of the antecedents of relapse and the treatment measures that can be taken to prevent or limit relapse after treatment completion. This relapse prevention (RP) model, which was developed by Marlatt and Gordon (1985) and which has been widely used in recent years, has been the focus of considerable research. This article reviews various immediate and covert triggers of relapse proposed by the RP model, as well as numerous specific and general intervention strategies that may help patients avoid and cope with relapse-inducing situations. The article also presents studies that have provided support for the validity of the RP model.

## Overview of the RP Model

Marlatt and Gordon’s (1985) RP model is based on social-cognitive psychology and incorporates both a conceptual model of relapse and a set of cognitive and behavioral strategies to prevent or limit relapse episodes (for a detailed description of the development, theoretical underpinnings, and treatment components of the RP model, see Dimeff and Marlatt 1998; Marlatt 1996; Marlatt and Gordon 1985). A central aspect of the model is the detailed classification (i.e., taxonomy) of factors or situations that can precipitate or contribute to relapse episodes. In general, the RP model posits that those factors fall into two categories: immediate determinants (e.g., high-risk situations, a person’s coping skills, outcome expectancies, and the abstinence violation effect) and covert antecedents (e.g., lifestyle imbalances and urges and cravings).

Treatment approaches based on the RP model begin with an assessment of the environmental and emotional characteristics of situations that are potentially associated with relapse (i.e., high-risk situations). After identifying those characteristics, the therapist works forward by analyzing the individual drinker’s response to these situations, as well as backward to examine the lifestyle factors that increase the drinker’s exposure to high-risk situations. Based on this careful examination of the relapse process, the therapist then devises strategies to target weaknesses in the client’s cognitive and behavioral repertoire and thereby reduce the risk of relapse.

### Immediate Determinants of Relapse

#### High-Risk Situations

**Figure 1:**
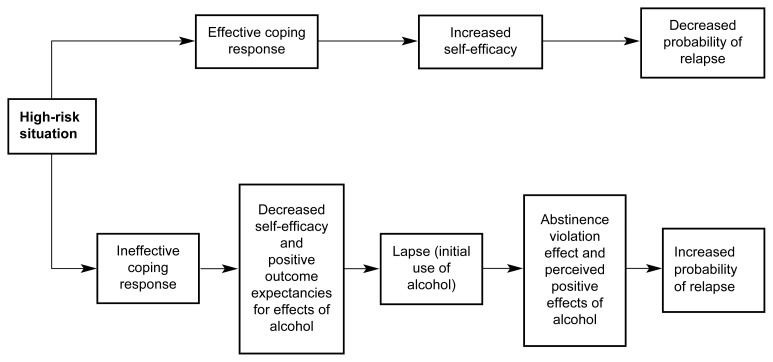
The cognitive-behavioral model of the relapse process posits a central role for high-risk situations and for the drinker’s response to those situations. People with effective coping responses have confidence that they can cope with the situation (i.e., increased self-efficacy), thereby reducing the probability of a relapse. Conversely, people with ineffective coping responses will experience decreased self-efficacy, which, together with the expectation that alcohol use will have a positive effect (i.e., positive outcome expectancies), can result in an initial lapse. This lapse, in turn, can result in feelings of guilt and failure (i.e., an abstinence violation effect). The abstinence violation effect, along with positive outcome expectancies, can increase the probability of a relapse. NOTE: This model also applies to users of drugs other than alcohol.

A central concept of the RP model postulates that high-risk situations frequently serve as the immediate precipitators of initial alcohol use after abstinence (see figure 1). According to the model, a person who has initiated a behavior change, such as alcohol abstinence, should begin experiencing increased self-efficacy or mastery over his or her behavior, which should grow as he or she continues to maintain the change. Certain situations or events, however, can pose a threat to the person’s sense of control and, consequently, precipitate a relapse crisis. Based on research on precipitants of relapse in alcoholics who had received inpatient treatment, Marlatt (1996) categorized the emotional, environmental, and interpersonal characteristics of relapse-inducing situations described by study participants. According to this taxonomy, several types of situations can play a role in relapse episodes, as follows:

- Negative emotional states, such as anger, anxiety, depression, frustration, and boredom, which are also referred to as intrapersonal high-risk situations, are associated with the highest rate of relapse (Marlatt and Gordon 1985). These emotional states may be caused by primarily intrapersonal perceptions of certain situations (e.g., feeling bored or lonely after coming home from work to an empty house) or by reactions to environmental events (e.g., feeling angry about an impending layoff at work).
- Situations that involve another person or a group of people (i.e., interpersonal high-risk situations), particularly interpersonal conflict (e.g., an argument with a family member), also result in negative emotions and can precipitate relapse. In fact, intra-personal negative emotional states and interpersonal conflict situations served as triggers for more than one-half of all relapse episodes in Marlatt’s (1996) analysis.
- Social pressure, including both direct verbal or nonverbal persuasion and indirect pressure (e.g., being around other people who are drinking), contributed to more than 20 percent of relapse episodes in Marlatt’s (1996) study.
- Positive emotional states (e.g., celebrations), exposure to alcohol-related stimuli or cues (e.g., seeing an advertisement for an alcoholic beverage or passing by one’s favorite bar), testing one’s personal control (i.e., using “willpower” to limit consumption), and nonspecific cravings also were identified as high-risk situations that could precipitate relapse.

#### Coping

Although the RP model considers the high-risk situation the immediate relapse trigger, it is actually the person’s *response* to the situation that determines whether he or she will experience a lapse (i.e., begin using alcohol). A person’s coping behavior in a high-risk situation is a particularly critical determinant of the likely outcome. Thus, a person who can execute effective coping strategies (e.g., a behavioral strategy, such as leaving the situation, or a cognitive strategy, such as positive self-talk) is less likely to relapse compared with a person lacking those skills. Moreover, people who have coped successfully with high-risk situations are assumed to experience a heightened sense of self-efficacy (i.e., a personal perception of mastery over the specific risky situation) (Bandura 1977; Marlatt et al. 1995, 1999; Marlatt and Gordon 1985). Conversely, people with low self-efficacy perceive themselves as lacking the motivation or ability to resist drinking in high-risk situations.

#### Outcome Expectancies

Research among college students has shown that those who drink the most tend to have higher expectations regarding the positive effects of alcohol (i.e., outcome expectancies) and may anticipate only the immediate positive effects while ignoring or discounting the potential negative consequences of excessive drinking (Carey 1995). Such positive outcome expectancies may become particularly salient in high-risk situations, when the person expects alcohol use to help him or her cope with negative emotions or conflict (i.e., when drinking serves as “self-medication”). In these situations, the drinker focuses primarily on the anticipation of immediate gratification, such as stress reduction, neglecting possible delayed negative consequences.

#### The Abstinence Violation Effect

A critical difference exists between the first violation of the abstinence goal (i.e., an initial lapse) and a return to uncontrolled drinking or abandonment of the abstinence goal (i.e., a full-blown relapse). Although research with various addictive behaviors has indicated that a lapse greatly increases the risk of eventual relapse, the progression from lapse to relapse is not inevitable.

Marlatt and Gordon (1980, 1985) have described a type of reaction by the drinker to a lapse called the abstinence violation effect, which may influence whether a lapse leads to relapse. This reaction focuses on the drinker’s emotional response to an initial lapse and on the causes to which he or she attributes the lapse. People who attribute the lapse to their own personal failure are likely to experience guilt and negative emotions that can, in turn, lead to increased drinking as a further attempt to avoid or escape the feelings of guilt or failure. Furthermore, people who attribute the lapse to stable, global, internal factors beyond their control (e.g., “I have no willpower and will never be able to stop drinking”) are more likely to abandon the abstinence attempt (and experience a full-blown relapse) than are people who attribute the lapse to their inability to cope effectively with a specific high-risk situation. In contrast to the former group of people, the latter group realizes that one needs to “learn from one’s mistakes” and, thus, they may develop more effective ways to cope with similar trigger situations in the future.

### Covert Antecedents of High-Risk Situations

**Figure 2:**
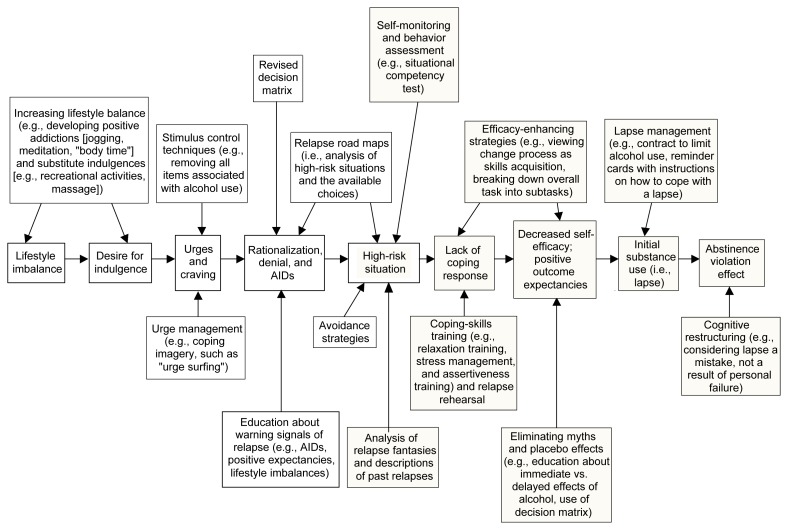
Covert antecedents and immediate determinants of relapse and intervention strategies for identifying and preventing or avoiding those determinants. Lifestyle balance is an important aspect of preventing relapse. If stressors are not balanced by sufficient stress management strategies, the client is more likely to use alcohol in an attempt to gain some relief or escape from stress. This reaction typically leads to a desire for indulgence that often develops into cravings and urges. Two cognitive mechanisms that contribute to the covert planning of a relapse episode—rationalization and denial—as well as apparently irrelevant decisions (AIDs) can help precipitate high-risk situations, which are the central determinants of a relapse. People who lack adequate coping skills for handling these situations experience reduced confidence in their ability to cope (i.e., decreased self-efficacy). Moreover, these people often have positive expectations regarding the effects of alcohol (i.e., outcome expectancies). These factors can lead to initial alcohol use (i.e., a lapse), which can induce an abstinence violation effect that, in turn, influences the risk of progressing to a full relapse. Self-monitoring, behavior assessment, analyses of relapse fantasies, and descriptions of past relapses can help identify a person’s high-risk situations. Specific intervention strategies (e.g., skills training, relapse rehearsal, education, and cognitive restructuring) and general strategies (e.g., relaxation training, stress management, efficacy-enhancing imagery, contracts to limit the extent of alcohol use, and reminder cards) can help reduce the impact of relapse determinants. Shaded boxes indicate steps in the relapse process and intervention measures that are specific to each client and his or her ability to cope with alcohol-related situations. White boxes indicate steps in the relapse process and intervention strategies that are related to the client’s general lifestyle and coping skills. High-risk situations are related to both the client’s general and specific coping abilities.

Although high-risk situations can be conceptualized as the immediate determinants of relapse episodes, a number of less obvious factors also influence the relapse process. These covert antecedents include lifestyle factors, such as overall stress level, as well as cognitive factors that may serve to “set up” a relapse, such as rationalization, denial, and a desire for immediate gratification (i.e., urges and cravings) (see figure 2). These factors can increase a person’s vulnerability to relapse both by increasing his or her exposure to high-risk situations and by decreasing motivation to resist drinking in high-risk situations.

In many cases, initial lapses occur in high-risk situations that are completely unexpected and for which the drinker is often unprepared. In relapse “set ups,” however, it may be possible to identify a series of covert decisions or choices, each of them seemingly inconsequential, which in combination set the person up for situations with overwhelmingly high risk. These choices have been termed “apparently irrelevant decisions” (AIDs), because they may not be overtly recognized as related to relapse but nevertheless help move the person closer to the brink of relapse. One example of such an AID is the decision by an abstinent drinker to purchase a bottle of liquor “just in case guests stop by.” Marlatt and Gordon (1985) have hypothesized that such decisions may enable a person to experience the immediate positive effects of drinking while disavowing personal responsibility for the lapse episode (“How could anyone expect me not to drink when there’s a bottle of liquor in the house?”).

#### Lifestyle Factors

Marlatt and Gordon (1985) have proposed that the covert antecedent most strongly related to relapse risk involves the degree of balance in the person’s life between perceived external demands (i.e., “shoulds”) and internally fulfilling or enjoyable activities (i.e., “wants”). A person whose life is full of demands may experience a constant sense of stress, which not only can generate negative emotional states, thereby creating high-risk situations, but also enhances the person’s desire for pleasure and his or her rationalization that indulgence is justified (“I owe myself a drink”). In the absence of other non-drinking pleasurable activities, the person may view drinking as the only means of obtaining pleasure or escaping pain.

#### Urges and Cravings

The desire for immediate gratification can take many forms, and some people may experience it as a craving or urge to use alcohol. Although many researchers and clinicians consider urges and cravings primarily physiological states, the RP model proposes that both urges and cravings are precipitated by psychological or environmental stimuli. Ongoing cravings, in turn, may erode the client’s commitment to maintaining abstinence as his or her desire for immediate gratification increases. This process may lead to a relapse setup or increase the client’s vulnerability to unanticipated high-risk situations.

Although they are often used interchangeably, the terms “urges” and “cravings” can be associated with distinct meanings. Thus, Marlatt and Gordon (1985) have defined an urge as a relatively sudden impulse to engage in an act such as alcohol consumption, whereas craving is defined as the subjective desire to experience the effects or consequences of such an act. Nevertheless, the same processes may mediate both urges and cravings. Two such processes have been proposed: (1) conditioning^1^ elicited by stimuli associated with past gratification and (2) cognitive processes associated with anticipated gratification (i.e., the expectancies for the immediate pleasurable effects of alcohol).

## RP Intervention Strategies

The RP model includes a variety of cognitive and behavioral approaches designed to target each step in the relapse process (see figure 2). These approaches include specific intervention strategies that focus on the immediate determinants of relapse as well as global self-management strategies that focus on the covert antecedents of relapse. Both the specific and global strategies fall into three main categories: skills training, cognitive restructuring, and lifestyle balancing.

### Specific Intervention Strategies

The goal of the specific intervention strategies—identifying and coping with high-risk situations, enhancing self-efficacy, eliminating myths and placebo effects, lapse management, and cognitive restructuring—is to teach clients to anticipate the possibility of relapse and to recognize and cope with high-risk situations. These strategies also focus on enhancing the client’s awareness of cognitive, emotional, and behavioral reactions in order to prevent a lapse from escalating into a relapse. The first step in this process is to teach clients the RP model and to give them a “big picture” view of the relapse process. For example, the therapist can use the metaphor of behavior change as a journey that includes both easy and difficult stretches of highway and for which various “road signs” (e.g., “warning signals”) are available to provide guidance. According to this metaphor, learning to anticipate and plan for high-risk situations during recovery from alcoholism is equivalent to having a good road map, a well-equipped tool box, a full tank of gas, and a spare tire in good condition for the journey.

#### Identifying and Coping With High-Risk Situations

To anticipate and plan accordingly for high-risk situations, the person first must identify the situations in which he or she may experience difficulty coping and/or an increased desire to drink. These situations can be identified using a variety of assessment strategies. For example, the therapist can interview the client about past lapses or relapse episodes and relapse dreams or fantasies in order to identify situations in which the client has or might have difficulty coping. Several self-report questionnaires also can help assess the situations in which clients have been prone to drinking heavily in the past as well as the clients’ self-efficacy for resisting future drinking in these situations (Annis and Davis 1988; Annis 1982*a*). Furthermore, clients who have not yet initiated abstinence are encouraged to self-monitor their drinking behavior—for example, by maintaining an ongoing record of the situations, emotions, and interpersonal factors associated with drinking or urges to drink. Such a record allows clients to become more aware of the immediate precipitants of drinking. Even in clients who have already become abstinent, self-monitoring can still be used to assess situations in which urges are more prevalent.

Once a person’s high-risk situations have been identified, two types of intervention strategies can be used to lessen the risks posed by those situations. The first strategy involves teaching the client to recognize the warning signals associated with imminent danger—that is, the cues indicating that the client is about to enter a high-risk situation. Such warning signals to be recognized may include, for example, AIDs, stress and lack of lifestyle balance, and strong positive expectances about drinking. As a result of identifying those warning signals, the client may be able to take some evasive action (e.g., escape from the situation) or possibly avoid the high-risk situation entirely.

The second strategy, which is possibly the most important aspect of RP, involves evaluating the client’s existing motivation and ability to cope with specific high-risk situations and then helping the client learn more effective coping skills. Relevant coping skills can be behavioral or cognitive in nature and can include both strategies to cope with specific high-risk situations (e.g., refusing drinks in social situations and assertive communication skills) and general strategies that can improve coping with various situations (e.g., meditation, anger management, and positive self-talk).

Assessing a client’s existing coping skills can be a challenging task. Questionnaires such as the situational confidence test (Annis 1982*b*) can assess the amount of self-efficacy a person has in coping with drinking-risk situations. Those measures do not necessarily indicate, however, whether a client is actually able or willing to use his or her coping skills in a high-risk situation. To increase the likelihood that a client can and will utilize his or her skills when the need arises, the therapist can use approaches such as role plays and the development and modeling of specific coping plans for managing potential high-risk situations.

#### Enhancing Self-Efficacy

Another approach to preventing relapse and promoting behavioral change is the use of efficacy-enhancement procedures—that is, strategies designed to increase a client’s sense of mastery and of being able to handle difficult situations without lapsing. One of the most important efficacy-enhancing strategies employed in RP is the emphasis on collaboration between the client and therapist instead of a more typical “top down” doctor-patient relationship. In the RP model, the client is encouraged to adopt the role of colleague and to become an objective observer of his or her own behavior. In developing a sense of objectivity, the client is better able to view his or her alcohol use as an addictive behavior and may be more able to accept greater responsibility both for the drinking behavior and for the effort to change that behavior. Clients are taught that changing a habit is a process of skill acquisition rather than a test of one’s willpower. As the client gains new skills and feels successful in implementing them, he or she can view the process of change as similar to other situations that require the acquisition of a new skill.

Another efficacy-enhancing strategy involves breaking down the overall task of behavior change into smaller, more manageable subtasks that can be addressed one at a time (Bandura 1977). Thus, instead of focusing on a distant end goal (e.g., maintaining lifelong abstinence), the client is encouraged to set smaller, more manageable goals, such as coping with an upcoming high-risk situation or making it through the day without a lapse. Because an increase in self-efficacy is closely tied to achieving preset goals, successful mastery of these individual smaller tasks is the best strategy to enhance feelings of self-mastery.

Therapists also can enhance self-efficacy by providing clients with feedback concerning their performance on other new tasks, even those that appear unrelated to alcohol use. In general, success in accomplishing even simple tasks (e.g., showing up for appointments on time) can greatly enhance a client’s feelings of self-efficacy. This success can then motivate the client’s effort to change his or her pattern of alcohol use and increase the client’s confidence that he or she will be able to successfully master the skills needed to change.

#### Eliminating Myths and Placebo Effects

Counteracting the drinker’s misperceptions about alcohol’s effects is an important part of relapse prevention. To accomplish this goal, the therapist first elicits the client’s positive expectations about alcohol’s effects using either standardized questionnaires or clinical interviews. Positive expectancies regarding alcohol’s effects often are based on myths or placebo effects of alcohol (i.e., effects that occur because the drinker expects them to, not because alcohol causes the appropriate physiological changes). In particular, considerable research has demonstrated that alcohol’s perceived positive effects on social behavior are often mediated by placebo effects, resulting from both expectations (i.e., “set”) and the environment (i.e., “setting”) in which drinking takes place (Marlatt and Rohsenow 1981). Subsequently, the therapist can address each expectancy, using cognitive restructuring (which is discussed later in this section) and education about research findings. The therapist also can use examples from the client’s own experience to dispel myths and encourage the client to consider both the immediate and the delayed consequences of drinking.

Even when alcohol’s perceived positive effects are based on actual drug effects, often only the immediate effects are positive (e.g., euphoria), whereas the delayed effects are negative (e.g., sleepiness), particularly at higher alcohol doses. Asking clients questions designed to assess expectancies for both immediate and delayed consequences of drinking versus not drinking (i.e., using a decision matrix) (see table, p. 157) often can be useful in both eliciting and modifying expectancies. With such a matrix, the client can juxtapose his or her own list of the delayed negative consequences with the expected positive effects.

#### Lapse Management

Despite precautions and preparations, many clients committed to abstinence will experience a lapse after initiating abstinence. Lapse-management strategies focus on halting the lapse and combating the abstinence violation effect to prevent an uncontrolled relapse episode. Lapse management includes contracting with the client to limit the extent of use, to contact the therapist as soon as possible after the lapse, and to evaluate the situation for clues to the factors that triggered the lapse. Often, the therapist provides the client with simple written instructions to refer to in the event of a lapse. These instructions reiterate the importance of stopping alcohol consumption and (safely) leaving the lapse-inducing situation. Lapse management is presented to clients as an “emergency preparedness” kit for their “journey” to abstinence. Many clients may never need to use their lapse-management plan, but adequate preparation can greatly lessen the harm if a lapse does occur.

#### Cognitive Restructuring

Cognitive restructuring, or reframing, is used throughout the RP treatment process to assist clients in modifying their attributions for and perceptions of the relapse process. In particular, cognitive restructuring is a critical component of interventions to lessen the abstinence violation effect. Thus, clients are taught to reframe their perception of lapses—to view them not as failures or indicators of a lack of willpower but as mistakes or errors in learning that signal the need for increased planning to cope more effectively in similar situations in the future. This perspective considers lapses key learning opportunities resulting from an interaction between coping and situational determinants, both of which can be modified in the future. This reframing of lapse episodes can help decrease the clients’ tendency to view lapses as the result of a personal failing or moral weakness and remove the self-fulfilling prophecy that a lapse will inevitably lead to relapse.

### Global Lifestyle Self-Control Strategies

Although specific intervention strategies can address the immediate determinants of relapse, it is also important to modify individual lifestyle factors and covert antecedents that can increase exposure or reduce resistance to high-risk situations. Global self-control strategies are designed to modify the client’s lifestyle to increase balance as well as to identify and cope with covert antecedents of relapse (i.e., early warning signals, cognitive distortions, and relapse set-ups).

#### Balanced Lifestyle and Positive Addiction

Assessing lifestyle factors associated with increased stress and decreased lifestyle balance is an important first step in teaching global self-management strategies. This assessment can be accomplished through approaches in which clients self-monitor their daily activities, identifying each activity as a “want,” “should,” or combination of both. Clients also can complete standardized assessment measures, such as the Daily Hassles and Uplifts Scale (Delongis et al. 1982), to evaluate the degree to which they perceive their life stressors to be balanced by pleasurable life events.

Many clients report that activities they once found pleasurable (e.g., hobbies and social interactions with family and friends) have gradually been replaced by drinking as a source of entertainment and gratification. Therefore, one global self-management strategy involves encouraging clients to pursue again those previously satisfying, non-drinking recreational activities. In addition, specific cognitive-behavioral skills training approaches, such as relaxation training, stress-management, and time management, can be used to help clients achieve greater lifestyle balance.

Helping the client to develop “positive addictions” (Glaser 1976)—that is, activities (e.g., meditation, exercise, or yoga) that have long-term positive effects on mood, health, and coping—is another way to enhance lifestyle balance. Self-efficacy often increases as a result of developing positive addictions, largely caused by the experience of successfully acquiring new skills by performing the activity.

**Table:** An Example of a Decision Matrix for Alcohol Abstinence or Alcohol Use*

	Immediate Consequences		Delayed Consequences	
		
	Positive	Negative	Positive	Negative
		
**Remain Abstinent**	Improved self-efficacy and self-esteem, family approval, better health, more energy, save money and time, greater success at work	Frustration and anxiety, denied pleasures of drinking, unable to go to bars, anger at not being able to do what one wants without “paying the price”	Greater control over one’s life, better health and longevity, learn about one’s self and others without being intoxicated, more respect from others	Not able to enjoy drinking while watching sports, bored and depressed, not able to remain friends with heavy-drinking buddies
**Resume Alcohol Use**	Automatic pleasure, reduced stress and anxiety, not feel pain, not worry about one’s problems, able to enjoy sports and drink with buddies	Feel weak from drinking, risk of accidents and embarrassment, anger of wife and family, arrive late to or miss work, hangovers, waste money	Maintain friendships with drinking buddies, able to drink while watching sports, not have to cope with wife and family by staying out drinking	Possible loss of family and job, deterioration of health and early death, loss of nondrinking or light-drinking friends, ridicule by others, low self-esteem

* In such a matrix, the client lists both the positive and negative immediate and delayed consequences of remaining abstinent versus resuming drinking. This list can facilitate the client’s decisionmaking process regarding his or her future alcohol consumption.

#### Stimulus-Control Techniques

Although achieving a more balanced lifestyle may reduce the risk of cravings and urges to use alcohol, urges and cravings might still result from exposure to conditioned stimuli previously associated with drinking. Stimulus-control techniques are relatively simple but effective strategies that can be used to decrease urges and cravings in response to such stimuli, particularly during the early abstinence period. Simply stated, these techniques encourage the client to remove all items directly associated with alcohol use from his or her home, office, and car. This includes eliminating, at least temporarily, all alcohol supplies, including those typically kept for “guests,” as well as packing away wine or shot glasses, corkscrews, and similar items. Clients who used to hide or stash alcoholic beverages should make a concerted effort to remember and remove alcohol from all possible hiding places, because these hidden or forgotten bottles can serve as a powerful temptation when found “accidentally” after a period of sobriety.

Other, more subtle items that may serve as conditioned cues for drinking may include the favorite living room easy chair or the music the client typically listened to while unwinding in the evening with several of his or her favorite drinks. In these cases, a temporary change in seating or listening habits may be helpful while the client develops alternative coping strategies. Similarly, certain social events or other high-risk situations may have become associated with excessive drinking to such an extent that they may induce classically conditioned urges or cravings, particularly in the early stages of abstinence. Accordingly, approaches that provide the client with a range of avoidance strategies for turning down invitations, leaving risky situations, or otherwise avoiding problematic places or events also can serve as stimulus-control measures that may help prevent a lapse.

#### Urge-Management Techniques

Even with effective stimulus-control procedures in place and an improved lifestyle balance, most clients cannot completely avoid experiencing cravings or urges to drink. Therefore, an important aspect of the RP model is to teach clients to anticipate and accept these reactions as a “normal” conditioned response to an external stimulus. According to this approach, the client should not identify with the urge or view it as an indication of his or her “desire” to drink. Instead, the client is taught to label the urge as an emotional or physiological response to an external stimulus in his or her environment that was previously associated with heavy drinking, similar to Pavlov’s dog, which continued to salivate at the sound of a bell that had previously signaled food.

In one clinical intervention based on this approach, the client is taught to visualize the urge or craving as a wave, watching it rise and fall as an observer and not to be “wiped out” by it. This imagery technique is known as “urge surfing” and refers to conceptualizing the urge or craving as a wave that crests and then washes onto a beach. In so doing, the client learns that rather than building interminably until they become overwhelming, urges and cravings peak and subside rather quickly if they are not acted on. The client is taught not to struggle against the wave or give in to it, thereby being “swept away” or “drowned” by the sensation, but to imagine “riding the wave” on a surf board. Like the conceptualization of urges and cravings as the result of an external stimulus, this imagery fosters detachment from the urges and cravings and reinforces the temporary and external nature of these phenomena.

#### Relapse Road Maps

Finally, therapists can assist clients with developing relapse road maps—that is, cognitive-behavioral analyses of high-risk situations that emphasize the different choices available to clients for avoiding or coping with these situations as well as their consequences. Such a “mapping out” of the likely outcomes associated with different choices along the way can be helpful in identifying AIDs. For example, if arguments with a former spouse are a high-risk situation, the therapist can help the client map out several possible scenarios for interacting with the ex-spouse, including the likelihood of precipitating an argument in each scenario. The therapist can then help identify coping responses that can be used to avoid a lapse at each point in the interaction.

## Theoretical and Practical Support for the RP Model

Several studies over the past two decades have evaluated the reliability and predictive validity^2^ of the RP model as well as the efficacy of treatment techniques based on this model. One recent large-scale research effort assessing the RP model was the Relapse Replication and Extension Project (RREP), which was funded by the National Institute on Alcohol Abuse and Alcoholism (Lowman et al. 1996). This collaborative research project evaluated the reliability of raters’ categorizations of high-risk situations using Marlatt’s taxonomy and assessed whether a prior situation could predict future lapse episodes.

As described earlier in this article, the original relapse taxonomy sought to categorize the environmental or emotional stimuli associated with an initial return to drinking in order to enhance the long-term effectiveness of aversion therapy. The resulting taxonomy contained three levels of categorization of high-risk situations with increasing specificity to help clinicians obtain detailed information about the causes underlying each relapse episode. In the RREP study, researchers from three study sites were trained in coding relapse episodes. The researchers then coded key, or baseline, relapse episodes^3^ described by study participants entering treatment at the study sites. The study addressed three major issues, as follows:

- It determined the inter-rater reliability of the relapse episode coding—that is, whether different researchers coded a given relapse episode in an identical or similar manner.
- It evaluated whether the key relapse episodes predicted the types of relapse episodes that study participants reported after undergoing treatment (Maisto et al. 1996; Stout et al. 1996).
- It extended research on the RP model beyond the taxonomy by evaluating alternative methods for assessing high-risk situations as well as evaluating the relative contribution of negative affect, abstinence violation effect, coping, and expectancies on the likelihood of relapse.

The results reported in the RREP study indicate that the original relapse taxonomy of the RP model has only moderate inter-rater reliability at the highest level of specificity, although reliability of the more general categories (e.g., negative affect and social pressure) was better. The model’s predictive validity also was modest; however, the definition of the key relapse episodes utilized in these studies failed to clarify whether these were voluntary change episodes or simply a return to drinking following a short period of abstinence that did not represent a serious attempt to quit drinking. Therefore, the RREP studies do not represent a good test of the predictive validity of the taxonomy.

Nevertheless, the study provides relatively good support for other aspects of the RP model. For example, Miller and colleagues (1996) found that although mere exposure to specific high-risk situations did not predict relapse, the manner in which people coped with those situations strongly predicted subsequent relapse or continued abstinence. Furthermore, in that study the majority of relapse episodes after treatment occurred during situations involving negative emotional states, a finding that has been replicated in other studies (Cooney et al. 1997; McKay 1999; Shiffman 1992). Finally, the results of Miller and colleagues (1996) support the role of the abstinence violation effect in predicting which participants would experience a full-blown relapse following an initial lapse. Specifically, those participants who had a greater belief in the disease model of alcoholism and a higher commitment to absolute abstinence (who were most likely to experience feelings of guilt over their lapse) were most likely to experience relapse in that study. In a recent review of the literature on relapse precipitants, Dimeff and Marlatt (1998) also concluded that considerable support exists for the notion that an abstinence violation effect can precipitate a relapse.

Several recent review articles and meta-analyses have examined the effectiveness of treatments based on the RP model in preventing relapse (Dimeff and Marlatt 1998; Rawson et al. 1993; Carroll 1996; Irvin et al. 1999). The RP-based treatments included in those analyses were delivered both as stand-alone treatments for initiating abstinence and as adjuncts to other treatment programs. Although the reviews differ in their methodology and in their criteria for including or excluding certain treatments, the conclusions regarding overall effectiveness of the RP approach are similar. The findings can be summarized as follows:

- The studies conducted to date tend to support the effectiveness of cognitive-behavioral RP-based approaches in reducing the frequency of relapse episodes as well as the intensity of lapse and/or relapse episodes among people who resumed alcohol use after treatment (Irvin et al. 1999). The effectiveness of RP was particularly great in studies that compared relapse rates in patients before and after treatment or that compared patients receiving RP-based treatment with controls receiving no treatment.
- Despite its benefits, RP-based treatment is not associated with higher abstinence rates compared with other valid treatment approaches (Carroll 1996; Irvin et al. 1999). RP-based treatment is, however, often associated with lower drinking rates and fewer drinking problems among patients who have experienced a relapse (e.g., Chaney et al. 1978).
- RP is associated with “delayed emergence effects”—that is, significant effects favoring RP as compared with other treatment approaches are often found only at later followup points (i.e., 1 year or more after treatment) (Carroll 1996). This delayed effectiveness may result from the fact that it takes time to learn new skills and that consequently RP effects become more obvious as patients acquire additional practice.
- Although RP has been applied with some success to various addictive behaviors, the effects of RP-based approaches are greatest in the treatment of alcoholism or multiple drug use (Irvin et al. 1999).
- Combining RP with medications (e.g., disulfiram or naltrexone) to treat alcoholism leads to improved outcomes as compared with either RP or medication alone (Irvin et al. 1999).

## Summary

The RP model of relapse is centered around a detailed taxonomy of emotions, events, and situations that can precipitate both lapses and relapses to drinking. This taxonomy includes both immediate relapse determinants and covert antecedents, which indirectly increase a person’s vulnerability to relapse. Based on the classification of relapse determinants and high-risk situations proposed in the RP model, numerous treatment components have been developed that are aimed at helping the recovering alcoholic cope with high-risk situations. The results of recent research, particularly the RREP study, likely will lead to modifications of the original RP model, particularly with regard to the assessment of high-risk situations as well as the conceptualization of covert and immediate antecedents of relapse. Overall, however, research findings support both the overall model of the relapse process and the effectiveness of treatment strategies based on the model.

## Footnotes

1 Classical or Pavlovian conditioning occurs when an originally neutral stimulus (e.g., the sight of a beer bottle) is repeatedly paired with a stimulus (e.g., alcohol consumption) that induces a certain physiological response. After the two stimuli have been paired repeatedly, the neutral stimulus becomes a conditioned stimulus that elicits the same physiological response.

2 The term “reliability” refers to the ability of a test or method to provide stable results (e.g., when different patients are compared or different investigators rate the same patient). The term “predictive validity” refers to the ability of a test or method to predict a certain outcome (e.g., relapse risk) accurately.

3 The key relapse episode was defined as the most recent use of alcohol following at least 4 days of abstinence (Longabaugh et al. 1996).
